# How are handover delays from ambulances to emergency departments being addressed in the United Kingdom? A nationwide survey of ambulance services and emergency departments

**DOI:** 10.1186/s12873-026-01532-9

**Published:** 2026-03-27

**Authors:** Bárbara Santos Gomes, Isobel Joy McFadzean, Timothy Driscoll, Mark Kingston, Mari Jones, Natalie Joseph-Williams, Steve Goodacre, Sioned Gwyn, Ashrafunessa Khanom, Hilary Pillin, Helen Snooks, Andrew Carson-Stevens, Deborah Fitzsimmons

**Affiliations:** 1 Faculty of Medicine, Health and Life Science, Swansea University, Swansea, UK; 2 Division of Population Medicine, School of Medicine, Cardiff University, Cardiff, UK; 3 PRIME Centre Wales, Cardiff, UK; 4 Sheffield Centre for Health and Related Research, Sheffield, UK; 5 Association of Ambulance Chief Executives, London, UK

**Keywords:** Ambulance queuing, Ambulance service, Emergency department, Handover delays, Ambulance ramping, Ambulance offload delays, Crowding, Patient flow, Survey, Systems thinking

## Abstract

**Background:**

Excessive waiting times and ambulance handover delays are of high concern to healthcare professionals and the public internationally. Ambulance services and emergency departments (EDs) have attempted to mitigate delays but the initiatives implemented have not been systematically described. To inform site selection for a national evaluation of such initiatives (the STALLED study), we set out to identify and describe initiatives that have been implemented at the ED entrance to address delayed ambulance handover in the United Kingdom (UK).

**Methods:**

Survey of current practice in all UK ambulance services (a total of 13) using a semi-structured questionnaire, distributed by email, focusing on initiatives based at the door of emergency departments, for completion and return by email or telephone interview. We also sent the questionnaire to a purposive sample of 24 EDs, identified from ambulance service responses. We summarised and coded initiatives reported and mapped them to the Systems Engineering Initiative for Patient Safety model, to support an understanding of where and how those initiatives influenced the healthcare system.

**Results:**

Twelve of 13 ambulance services and 16 of 24 EDs responded to the questionnaire describing 34 and 36 initiatives respectively. All respondents reported having several (between two and 12 per service) initiatives in place to reduce handover delays, most commonly involving ambulance staff caring for groups of patients in ED corridors (8/12), coordinated patient handovers within a defined time period (7/12), and ED reconfiguration to facilitate rapid offload (10/16). Most initiatives focussed on changes which influenced the organisation of care, the introduction or revision of key tasks and roles for staff, as well as changes to the ED environment.

**Conclusions:**

Ambulance services and EDs have implemented a variety of initiatives to reduce handover delays. Most of the initiatives involve multiple parts of the system, including tasks, staff, the organisation and the internal environment. These complex initiatives require careful study to understand how they work and how they can inform best practice.

**Supplementary Information:**

The online version contains supplementary material available at 10.1186/s12873-026-01532-9.

## Background

Ambulance handover delays, also known as ambulance ramping and ambulance offload delays, are a pressing issue in many countries [1], with patients sometimes remaining in ambulances for several hours before being transferred into the hospital [2]. The wait may also occur after the patient has been moved from the ambulance into the emergency departments (EDs) but while still under the care of ambulance service staff, when insufficient ED staffing levels prevent handovers, or there are no suitable facilities within the department in which to leave the patient. During these delays, patients are deprived of ED care, and ambulances are unable to respond to new emergencies, creating widespread upstream and downstream “knock-on” effects for patients and staff throughout the urgent and emergency care system.

Whilst some hospitals experience high levels of ambulance handover delays, others manage to maintain minimal wait times. However, the reasons for these variations are not well understood. Little is known about what successful sustainable initiatives and mitigations are in place, issues in implementation, or what works to reduce handover delays, related harms (to patients [3] and staff [4, 5]) and costs [1]. Better understanding of this could help more services to reduce waiting times.

We know the origins of such delays are a systemwide problem, and changes to improve flow are needed in the ED and beyond. Hospital handover delays are caused by factors across the whole health system, including hospital capacity, patient discharge processes and bed occupancy and ‘a whole-system approach is needed to tackle them’ [6, 7]. Recent reviews of the global literature on ambulance handover delays indicate that research has predominantly focused on identifying causes and effects, and assessing proposed solutions, with ambulance diversion from overcrowded EDs emerging as the most frequently studied initiative – albeit with mixed outcomes [1, 8]. Other interventions led by ambulance services (ASs) include redirecting patients to alternative care destinations and nationally stipulated operational changes such as escalation processes to enable paramedics to handover within an agreed maximum time. Hospital-based initiatives include the implementation of offload zones, expanding ED capacity through additional beds and increasing ED patient throughput – such as fast-track systems, utility beds and overcapacity protocols - again with debatable results. A common theme across the reviewed literature was that this issue encompasses clinical, operational and administrative domains and therefore requires a system-wide approach for effective mitigation.

Ambulance handover delays have become an important policy concern in the United Kingdom (UK) [9]. An expectation of ambulance handover within 15 min of arrival at EDs was stipulated by the Department of Health and Social Care in England more than a decade ago [10]. In 2012, the Association of Ambulance Chief Executives (AACE) and the National Health Service (NHS) Confederation, both representative bodies for healthcare provider organisations in the UK, collaborated to produce a joint report focusing on eradicating handover delays, embracing a ‘zero tolerance’ approach to delays [10]. Despite this, delays in hospital handovers have continued to increase over the last decade for all ambulance services, particularly during and since the 2020–2022 COVID-19 pandemic [11]. More recently, some services have been able to significantly reduce handover times at ED, with their respective hospitals [12], but little is documented about what makes certain initiatives successful in reducing handover delays overall. A better understanding of this could help more services to meet the national 15-minute standard.

AACE has issued in recent years multiple reports focusing on handover delays, the harm caused by such delays [3] and key ambulance metrics impacted by these delays, while also promoting successful initiatives in hospitals across England, where leadership and culture were highlighted as prevalent themes [2, 12]. A scoping review of the effectiveness of 10 high-impact initiatives, identified by NHS England, for recovering urgent and emergency care services, indicated that the overall evidence base is limited in both quantity and quality, highlighting the need for evaluation of well-defined and specific initiatives [13]. 

Using a whole-system approach, the STALLED study [STALLED: What works to improve safety, patient experience, outcomes and costs related to delayed ambulance handovers at emergency departments? A whole system approach] aims to provide evidence about what works to improve safety, patient experience, outcomes and costs related to ambulance queuing [14]. To support site selection for subsequent phases of the STALLED study, we conducted a service evaluation by surveying ambulance services and EDs across the UK. We asked them to identify and describe initiatives that aim to reduce handover delays and related harms, notably to understand where and how they targeted different areas of the system.

## Methods

### Data collection

We used a cross-sectional survey methodology to conduct a brief semi-structured questionnaire for completion and return by email or telephone interview. The research team developed the questionnaire to partially address one of the study objectives - to identify and describe initiatives currently in use across the UK to reduce handover delays and related harms. We developed and piloted the questionnaire based on published literature [12] and expert stakeholder input from AACE, and ED and ambulance services leaders. Two closely aligned questionnaires were developed: one for AS participants and one for ED participants (see Supplementary Information A and B). The survey was designed to support site selection for subsequent phases of the STALLED study and to identify initiatives that could be characterised and studied, rather than provide a representative overview of UK practice. The questionnaires included closed and open-ended questions focusing on initiatives based in or at the door of EDs with a suggested categorisation (ED clinician care provided on ambulances, paramedic care within the ED, use of additional space, use of additional staff, other), key activities of each initiative and impact on the services and staff. The impact of these initiatives is not reported in this manuscript, as it falls beyond its intended scope. We followed a consensus-based checklist for reporting of survey studies [15]. 

BG, STALLED study manager and researcher, emailed the questionnaire to all UK ambulance services (a total of 13) on 12/08/2024, with fortnightly reminders, addressed to chief executives, quality directors and heads of research. BG also sent the questionnaire to a purposive sample of 24 hospital EDs (12 with high levels of delays; 12 with low levels) on 21/11/2024, with fortnightly reminders, and addressed to Hospital Chief Executives, ED directors and key contacts identified in the AS survey.

Hospital EDs were sampled based on the responses from the ASs to the following survey question: ‘Please identify 2–3 hospitals in your ambulance service region where levels of ambulance handover delays are high and 2–3 hospitals where levels of delays are low’, using their own definition. TD, STALLED study data manager and statistician, then validated ambulance service suggestions using publicly available ambulance handover data from NHS England for winter 2022/23 [16]. For Wales, we used equivalent data published at local health board level. NHS Scotland publish weekly handover data at hospital, health board, and national level comprising number of handovers, median handover time, and 90th centile handover time; as these were not directly comparable to England or Wales, we checked data for December 2022 only, and compared only with other hospitals in Scotland.

Hospitals were considered to have high or low delays if they were in the upper or lower quartile for ambulance time-lost-per-patient in their ambulance service area; precise thresholds were adjusted on a per-service basis to ensure hospitals with similar handover times were grouped consistently where possible. The first validated hospital in each category (low and high) suggested by each ambulance service was then selected for the survey.

Surveyed hospitals were located in England, Wales and Scotland. Due to the lack of a response from the AS and the absence of publicly available data on ambulance handover delays in Northern Ireland, we did not contact EDs located in that region.

### Analysis

BG logged the completed questionnaires and entered all responses into a spreadsheet for analysis. BG and HS discussed the responses and coded initiatives based on the original questionnaire categorisation, reclassifying responses provided where information was duplicated, overlapping or did not fit. SGw, an academic GP, read the questionnaire responses and the spreadsheet to ensure that all information was captured, incorporating quality assurance and data validation measures throughout the data compilation process.

To make sense of responses provided, we then used a Framework Analysis [17] approach based on the Systems Engineering Initiative for Patient Safety (SEIPS) domains [18]. The data were read and re-read by academic GPs with experience in human factors and patient safety, JM and ACS, to support data familiarisation. They summarised the initiatives and mapped them to the six SEIPS domains [18]: ‘tools and technology’ (accessibility, functionality and maintenance), ‘internal environment’ (physical environment characteristics), ‘tasks’ (actions within larger work processes), ‘organisation’ (time, space, resources and activity), ‘person’ (individual characteristics) and ‘external environment’ (societal, economic, regulatory and policy factors externally) [18]. BG then independently mapped initiatives to these domains to assess concordance. Following review, concordance was found between ACS, JM and BG with regards to the mapping of initiatives to domains. This process allowed for identification of the range of initiatives within, and across the SEIPS domains, supporting a structured approach to understanding how organisations focused on different components of the healthcare system to reduce handover delays.

## Results

We received responses from 12/13 ASs and from 16/24 EDs. The responding organisations were based in England (10 ASs and 13 EDs), Scotland (1 AS and 1 ED) and Wales (1 AS and 2 EDs). Of the 16 EDs, seven had low levels of ambulance handover delays (randomly coded ED1 to ED7), and nine had high levels of delays (randomly coded ED8 to ED16).

Responses were received by email between 15/08/2024 and 13/01/2025, except for one delayed response, which was received on 02/05/2025. One AS requested an online meeting to answer the questions. The occupation/role of questionnaire respondents included consultant paramedics, directors of operations, heads of patient flow, ED clinical leads, performance managers and ED lead matrons (see Supplementary Information, section C, for full details).

Mapping of participating ambulance services to their associated EDs, respective category of levels of delays (high or low), and bed capacity and occupancy, can be found in the Supplementary Information, section D. The total number of handovers in Winter 22/23 for each participating ambulance service is also detailed in the Supplementary Information, section E. These data are presented banded, to anonymise responding organisations.

All respondents reported having several (between two and 12) initiatives in place to reduce handover delays (see Table 1, below).

Initiatives are listed in order of frequency and included the provision of care by ambulance staff within the ED; rapid offload of patients to the ED after a defined period of time; reconfiguration of facilities or space to allow faster transfer of patients from ambulances to the ED; additional staff in the ED; additional multi-disciplinary assessment of patients in the ED; assessment of patients in the ambulance by ED staff; and various other role, infrastructure and workforce innovations.

**Table 1:** Initiatives reported by ASs and EDs

Type of initiative	Summary of initiatives with quotations included where they offer further detail or illumination	Mapping of initiatives to the SEIPS domains	Number of ASs (AS code) and EDs (ED code) that reported the initiative
Ambulance staff providing care within the ED (i1)	• “Cohorting” - Ambulance staff care for patients (up to 7 patients) in the ED corridors and ambulance foyer – sometimes the patient they brought to the ED, and sometimes also patients brought by other crews.	• Tasks • Person • Organisation • Internal environment	**8 ASs** (AS1-3, AS5-6, AS9-11) and **3 EDs** (ED6, ED11-12)
Rapid handover after agreed defined period (i2)	• Patients are handed over in the ED after escalation and an agreed defined period outside the ED. This was referred to by difference services as Timely Handover Process; Rapid Release; PUSH45; Power Hour. The set interval was reported as from 45–90 min. “They will either leave the patient on hospital trolley bed (if available) or an ambulance trolley bed that is already at ED” (AS3) • One AS reported an agreed process for immediate off-load to attend Immediately Life-Threatening calls.	• Tools and technology • Tasks • Person • Organisation • Internal environment • External environment	**7 ASs** (AS1, AS3-5, AS7, AS9, AS11) and **2 EDs** (ED4, ED16)
Infrastructure / space reconfiguration for rapid offload at ED (i3)	• New dedicated facilities for patients arriving by emergency ambulance (ambulance receiving centre / dedicated ambulance handover bay), dedicated chairs by the front door for those deemed to be ‘Fit2Sit’ for a rapid handover process; and increased capacity for ambulance offload, referred to by one service as “Patient on Ambulance Escalation Area: Ambulance assessment unit with dedicated nurse, healthcare assistant and senior clinician, and own dedicated ambulance entrance” • Move of disposal points from ED to ambulance entrance for quicker ambulance turnaround • Overnight use of daytime-only ward areas and using wards as temporary escalation spaces • Additional beds in corridors which was called ‘corridor care’	• Tools and technology • Tasks • Person • Organisation • Internal environment	**6 ASs** (AS1-2, AS4-5, AS9, AS12) and **10 EDs** (ED1, ED4-8, ED12-13, ED15-16)
Additional staff employed to co-ordinate flow/performance in ED (i4)	• Hospital Ambulance Liaison Officer (HALO) - offers oversight of the queue and supports crews with their time management, as well as highlighting barriers to effective handover as they arise; • Patient flow manager. • Duty Operational Manager.	• Tasks • Person • Organisation	**4 ASs** (AS1, AS6, AS8, AS10) **1 ED** (ED1)
Additional assessment by multidisciplinary team in ED (i5)	• Review at ED front door or on back of ambulances by multidisciplinary team e.g. Frailty team to redirect patients to community services e.g. virtual ward or admit patients directly to the ward from the ambulance; • “Multidisciplinary team attends ED to review ambulance cases to look for rapid discharge and admission avoidance.”	• Tasks • Person • Organisation • Internal environment	**3 ASs** (AS8-9, AS11)
ED staff providing assessment and treatment on ambulances (i6)	• ED staff, including medics (from residents to consultant grades) assess patients and nurses provide prescribed treatment to patients in ambulances in the queue to start earlier treatment. One service reported “ED clinician care provided on ambulances only happens in exceptional circumstances (triage).”	• Tasks • Person • Organisation • Internal environment	**3 ASs** (AS2, AS6, AS11) and **5 EDs** (ED1, ED4, ED8-9, ED16)
Innovations to speed up initial triage (i7)	• Redesign of the front door with increased capacity in the Rapid Assessment and Treatment team, such as additional administrative support; beds introduced into the rapid assessment area and streaming patients into other areas of the Trust, sometimes with a doctor stationed at the front door, or redirection to Same Day Emergency Care (SDEC) during rapid triage processes.	• Tools and technology • Tasks • Person • Organisation • Internal environment	**2 ASs** (AS11-12), and **1 ED** (ED16)
Improved communication through workflow rules, handover protocols or digital/visual mechanisms at ED (i8)	• Initiatives co-designed by hospitals and ASs such as traffic light systems, ambulance handover safety checklists and processes, pre-booking tasks by reception staff using ambulance services electronic patient report forms, escalation action cards, communication channels. • Removal of need for clinician-to-clinician handover for patients requiring an Urgent Treatment Centre (UTC) and relocation of UTC so that it is close to the ED handover bay. • Visual management board showing free spaces for patients. • Shortened the ambulance handover form on the ED electronic patient record. • Surging triage staff to meet escalation.	• Tools and technology • Tasks • Person • Organisation • Internal environment	**2 AS** (AS4, AS10) and **1 ED** (ED16)
Additional role and skills for paramedics (i9)	• Additional skills, development, exposure and enhanced pay for paramedics. This included a concept referred to as Trusted assessor - paramedics and other ambulance clinicians being ‘trusted’ to follow inclusion / exclusion criteria and take patients direct to medical Same Day Emergency Care (SDEC). • Ambulance staff putting wrist bands on patients to assist identification for patient safety.	• Tools and technology • Tasks • Person • Organisation • Internal environment	**2 ASs** (AS6, AS9) and **1 ED** (ED7)
Workforce role redesign and deployment at ED (i10)	• Dedicated nurse for ambulance handovers, referred to as ‘Major Assessment Nurse (MAN)’ by one ED. • Clinical coordinator role - redirecting ambulance patients. • Change in triage nurse’s role and / or relocation closer to the ‘Hospital Arrival Screen’ (HAS) or receptionist for a quick handover. • Splitting the workload between the nurse in charge and handover nurse roles. • Oversight from Emergency Physician in Charge (EPIC) and / or the Emergency Nurse in Charge (ENIC) for early interventions (including sitting patients in the waiting room which are suitable and fall outside the AS criteria for ‘Fit2Sit’). • Use of a healthcare assistant to move patients to treatment areas and administration for booking in patients to free up ambulance crews. • Additional nursing and medical staff employed by the ED.	• Tasks • Person • Organisation • Internal environment	**1 AS** (AS4) and **8 EDs** (ED1-2, ED5-7, ED10, ED12-13)
New protocol for maintaining safety and quality of care when delays occur (i11)	• Minimum standard care policy - set of actions undertaken when delays occur. • Ambulance offload policy - principles used by ED staff in deciding which ambulance patient gets the next available Majors space. • Patients on ambulances are prioritised as if inside the ED. “Stable ambulance patients are booked in at reception on arrival, triaged by the Majors triage nurse, undergo our Rapid Assessment and Treatment process and imaging investigations, then return to the ambulance if no space has been identified during these processes”. (ED13) • “Majors in Minors” policy – “Patients who cannot access trollies in Majors, may be placed in waiting rooms, corridor chairs or clinical spaces within Minors (with or without trollies). The “Majors in Minors” policy provides staff with a framework for staff to escalate the needs of patients in the Minors area and to monitor the clinical risks being held there.” (ED13)	• Tasks • Organisation • Internal environment • External environment	**1 AS** (AS2) and **1 ED** (ED13)
Changes in discharge processes and space (i12)	• Discharge lounge open at weekends. • Transfer hubs created for patients awaiting departure from ED.	• Tasks • Organisation • Internal environment	**2 EDs** (ED14-15)

Other initiatives were reported that did not meet our inclusion criteria as they were not based in or at the door of ED, despite having wider implications for the health system. We summarise these below, but not frequency as we are aware these may exist more widely but were not reported by other services as they were out of scope; for example:

- **Ambulance service-based co-ordination to reduce delays across receiving hospitals**. Various initiatives based in the Ambulance Service were reported, including Tactical Operations Centre – 24/7 service monitoring hospital delays and escalating where required; tactical officer role; locality operational cell manager; ambulance capacity managers redirect patients away from one hospital site to another hospital site with less congestion (termed “Intelligent conveyance”);
- **Regular meetings to enhance communication**,** collaboration and staff engagement**. One service reported “Clinical Operational Huddle – twice weekly – a meeting of senior managers / directors to discuss clinical operations issued including hospital delays”;
- **‘Call before convey’ arrangements**. Professional-to-professional call (sometimes with an ED consultant or with senior clinician in a care coordination hub e.g. advanced practitioner or GP) to assist with decision support. Services reported Consultant Connect (telemedicine advice provider), single point of contact phone line, flow centre, frailty line, care coordination hubs;
- **Additional beds in wards**; and,
- **Ambulance crews ‘pre-alert’ by calling ahead of arrival to ED for time critical and immediate life-threatening calls** to let ED staff know and give the ability to clinical prepare for a patients arrival (Resus priority calls).

### SEIPS framework analysis

The mapping of initiatives to SEIPS allowed an exploratory descriptive analysis of where different domains influenced different areas of the health system as a whole, and were found to impact across the whole health system, although activities (or changes made in practice to improve outcomes) in some domains featured more frequently than others. A review across all domains can be found within Table 1 (Supplementary Information, section F).

For both ambulance services (33 of 34 initiatives) and emergency departments (31 of 36 initiatives), ‘Organisational’ initiatives occurred most frequently, focusing on time, space, resources and activity within the ED. These included changes to healthcare professional roles within the AS or ED; for example implementing an “Ambulance’ triage nurse focused on handovers”, and also new polices, such as for those ‘fit to sit’ within ED, ensuring that patients who were assessed as suitable, would be offloaded into needs-appropriate bays.

The next most frequently mapped domain was ‘Tasks’, or actions within larger processes for AS (25 of 34 initiatives) and for ED (25 of 36 initiatives). These included initiatives such as ambulance crews putting wristbands on patients to enhance patient identification and safety. These wristbands include accurate details that identify and match patients to their care.

## Discussion

This study is the first to provide a snapshot of initiatives in place across England, Scotland and Wales to reduce ambulance handover delays, focusing on initiatives based in or at the door of emergency departments. Specifically, we present a comprehensive overview of initiatives reported by ambulance services, supplemented by data from a convenience sample of emergency departments.

The response rate from the EDs was lower than that from ambulance services, though still within an acceptable range for survey-based research [19]. While ambulance handover delays are highly relevant to ambulance services due to their major impact on emergency response and patient safety, for EDs, these delays represent just one aspect of the broader challenge of patient flow [20]. ED crowding and patient flow are not specific to patients arriving by ambulance. As such, EDs must balance this issue with multiple other operational priorities, which may have influenced participating rates.

We iteratively developed a typology of initiatives. Some were reported more frequently than others, with cohorting in corridors, and coordinating rapid patient handovers after a defined period, being the most mentioned by the ambulance services. The use of such initiatives raises safety concerns [21] and they have not been fully evaluated although the initiatives are implemented with supporting guidance, developed collaboratively, intended to mitigate risks. Most of the EDs reported reconfiguration of their infrastructure or clinical workspace to aid rapid patient offloading.

Compared with existing literature [1, 7, 8], largely focused on identifying causes and effects of ambulance handover delays, or on evaluating discrete initiatives, our findings highlight a broader and more integrated perspective emerging from practice, reflecting coordinated adaptations across the care pathway. This study confirms that strategies previously described in the literature [1] - such as the use of offload zones, expansion of ED capacity and operational changes allowing paramedics to handover within an agreed maximum time – are being implemented across England, Scotland and Wales. In addition, we have identified a wide range of further strategies currently in use. These include, among others, ambulance staff providing care within the ED (including cohorting in corridors) and ED staff providing assessment and treatment to patients remaining in ambulances awaiting offload. This suggests an overlap of role and space between ambulance services and EDs, consistent with other studies that identify the blurring of role boundaries in emergency settings [22, 23]. 

Nearly all initiatives from both ambulance services and EDs could be mapped across all six SEIPS domains, which suggests a whole systems approach is being considered through initiatives being implemented to tackle handover delays. Most of the reported initiatives require changes in multiple parts of the system – staff, tasks, organisational and environment. These changes are complex, highlighting the need for thorough consideration of how to study them in order to understand how they work and can be adapted elsewhere.

Some emerging practices, such as additional assessment by multidisciplinary teams in ED (Table 1, i5), highlight greater opportunities for health system models of alternative pathways. These pathways allow patients to receive appropriate care at home or in the community setting. Patients may also be taken directly to a specialist service, e.g. a frailty unit, without the need for conveyance to EDs when appropriate, i.e. as identified through out-of-hospital multidisciplinary care coordination hubs. While beyond the scope of this study, some respondents reported initiatives aimed at addressing ambulance handover delays that were not based in or at the door of EDs, highlighting that broader system changes are occurring. Some of them align with existing literature [1, 8], and include shifting to clinical models providing care closer to home and using alternative care pathways to reduce unnecessary conveyance to ED.

Further research is required to generate robust evidence on what works to sustainably reduce handover delays and related harms, to inform best practices, and to enhance patient safety. Within subsequent phases of the STALLED study, we will be using findings from this survey to help guide our qualitative work packages. We will draw on the SEIPS domains to prompt perceptions and knowledge about the influence on handover delays focusing on a whole system approach. For instance, we will explore through in-depth interviews how policy, funding and external protocols influence these initiatives, which was mostly absent within the responses. Findings from the STALLED study will help us produce guidance on effective strategies for reducing delayed handovers. We will (i) investigate what policies and practices are being used in hospitals where ambulance queuing is rare, (ii) assess impact of successful queue management on patient flows, safety, experience, health and costs, and (iii) predict wider impacts of initiatives on patient flow through emergency care.

### Limitations

These initiatives stemmed mainly from an ambulance service perspective, with a smaller sample of EDs providing their perspective, focusing mainly on the interface between the ED and the ambulance service. Even though we contacted the four nations of the UK, a perspective from Northern Ireland has not been included in this study. Our sampling strategy may have missed important initiatives in unsampled EDs. The ED survey was not intended to be representative but to identify initiatives that could be characterised and studied. In addition, a structured questionnaire limits the depth of responses.

When analysing the responses, we observed that various organisations used different terminology. For instance, some services referred to ‘Timely Handover Process’, and it was also called ‘Rapid Release’, ‘PUSH45’ or ‘Power Hour’ by others – with all terms referring to rapid handover after a specified period. We have grouped these initiatives together based on their descriptions, without seeking further clarification from the respondents. Subsequent phases of the STALLED study will help contextualise and clarify what has been described within this initial scoping activity.

## Conclusions

Ambulance services and EDs have implemented a variety of initiatives aimed at reducing ambulance handover delays, including ambulance staff caring for groups of patients in the ED, coordinating patient handover within a defined period of time, and ED reconfiguration to facilitate rapid offload. When mapping the initiatives across the six domains of the SEIPS model, adopting a human factors and ergonomics approach, it was evident that most initiatives are diverse and complex in nature, involving changes in multiple parts of the system, including tasks, staff, the organisation and the internal environment. These complex initiatives require careful examination to understand how they work and how they can inform best practice. Evidence is urgently needed to understand the costs and effects of these initiatives on ambulance handover delays, patient outcomes, staff morale and the wider system.

## Supplementary Information

Below is the link to the electronic supplementary material.

Supplementary Material 1

## Data Availability

Anonymised questionnaire responses, collected and analysed during the current study, are available from the corresponding author on reasonable request.

## Abbreviations

AACE: Association of Ambulance Chief Executives
AS: Ambulance service
ED: Emergency Department
GP: General Practitioner
HALO: Hospital Ambulance Liaison Officer
NHS: National Health Service
SEIPS: System Engineering Initiative for Patient Safety
SDEC: Same Day Emergency Care
UK: United Kingdom
