# Supplementary material for: How are handover delays from ambulances to emergency departments being addressed in the United Kingdom? A nationwide survey of ambulance services and emergency departments
Source: BMC Emerg Med. 2026 Mar 27;26:134. doi: 10.1186/s12873-026-01532-9 (PMC13147741; doi:10.1186/s12873-026-01532-9)
Supplement: Supplementary file 1 — Supplementary Material 1 [file 12873_2026_1532_MOESM1_ESM.docx]

**How are handover delays from ambulances to emergency departments being addressed in the United Kingdom? A nationwide survey of ambulance services and emergency departments.**

**Supplementary Information**

1. **Questionnaire sent to ambulance services**

**STALLED - Survey of UK emergency ambulance services**

This is the first stage of the STALLED study. For further information, clarifications or to complete the questionnaire by telephone, please email Dr Barbara Gomes [b.gomes@swansea.ac.uk](mailto:b.gomes@swansea.ac.uk)

Your name:

Your job title:

Your e-mail address:

Date of completion:

Many thanks for supporting this work. If you would like to be added to the study mailing list, to be kept updated on study progress, please put an X here: _________

A1. Please identify 2-3 hospitals in your ambulance service region where levels of ambulance handover delays are

**High**

Hospital:

Hospital:

Hospital:

**Low**

Hospital:

Hospital:

Hospital:

Please use the following table to identify initiatives within your ambulance service area that aim to reduce handover delays at Emergency Departments. We specifically want to identify initiatives based in or at the doors of the ED.

|  | Initiative 1 | Initiative 2 | Initiative 3 |
| --- | --- | --- | --- |
| 1. Briefly describe the initiative | ED clinician care provided on ambulances  Paramedic care within the ED  Use of additional space  Use of additional staff  Other. Please specify: | ED clinician care provided on ambulances  Paramedic care within the ED  Use of additional space  Use of additional staff  Other. Please specify: | ED clinician care provided on ambulances  Paramedic care within the ED  Use of additional space  Use of additional staff  Other. Please specify: |
| 1. At which hospital is the initiative based? |  |  |  |
| 1. When was the initiative introduced to your service / hospital? |  |  |  |
| 1. What are the key activities of this initiative? |  |  |  |
| 1. How does this initiative affect the ambulance service or its staff? |  |  |  |
| 1. Is the impact of this initiative being measured? If yes, how? | yes no  How: |  |  |
| 1. Key contact in ED for further information | Name  Job title (if known)  Organisation  E-mail | Name  Job title (if known)  Organisation  E-mail | Name  Job title (if known)  Organisation  E-mail |

|  | Initiative 4 | Initiative 5 | Initiative 6 |
| --- | --- | --- | --- |
| 1. Briefly describe the initiative | ED clinician care provided on ambulances  Paramedic care within the ED  Use of additional space  Use of additional staff  Other. Please specify: | ED clinician care provided on ambulances  Paramedic care within the ED  Use of additional space  Use of additional staff  Other. Please specify: | ED clinician care provided on ambulances  Paramedic care within the ED  Use of additional space  Use of additional staff  Other. Please specify: |
| 1. At which hospital is the initiative based? |  |  |  |
| 1. When was the initiative introduced to your service/hospital? |  |  |  |
| 1. What are the key activities of this initiative? |  |  |  |
| 1. How does this initiative affect the ambulance service or its staff? |  |  |  |
| 1. Is the impact of this initiative being measured? If yes, how? | yes no  How: |  |  |
| 1. Key contact in ED for further information | Name  Job title (if known)  Organisation  E-mail | Name  Job title (if known)  Organisation  E-mail | Name  Job title (if known)  Organisation  E-mail |

1. **Questionnaire sent to Emergency Departments**

| Please use the table to identify initiatives **based in or at the doors of your Emergency Department** that aim to reduce ambulance/hospital handover delays. | | | |
| --- | --- | --- | --- |
|  | Initiative 1 | Initiative 2 | Initiative 3 |
| 1.Briefly describe the initiative | ED clinician care provided on ambulances  Paramedic care within the ED  Use of additional space  Use of additional staff  Other staff related changes  Changes in handover/discharge process/es  Other. Please specify: | ED clinician care provided on ambulances  Paramedic care within the ED  Use of additional space  Use of additional staff  Other staff related changes  Changes in handover/discharge process/es  Other. Please specify: | ED clinician care provided on ambulances  Paramedic care within the ED  Use of additional space  Use of additional staff  Other staff related changes  Changes in handover/discharge process/es  Other. Please specify: |
| 2. Was this initiative a formal policy/process change within the hospital/ department? | Yes  No |  |  |
| 3. When was the initiative introduced to this ED? |  |  |  |
| 4. What are the key activities of this initiative? |  |  |  |
| 5. How does this initiative affect the ED or its staff? |  |  |  |
| 6.How does this initiative affect patients? |  |  |  |
| 7. How often is this initiative used? (number of times per day/week; in certain occasions - please specify) |  |  |  |
| 8.Is the impact of this initiative being measured? If yes, how? | yes no  If yes, how: |  |  |

1. **Occupation/role of questionnaire respondents**

Ambulance services: Clinical Operations – Business Support Manager, Director of Operations, Senior Research Fellow, Lead Consultant Paramedic and Director for Care Quality & Professional Development, Clinical Team Educator & Paramedic, Team Leader (Operations), Operating Unit Manager, Assistant Director, Commissioning and Performance, Head of Patient Flow, Consultant Paramedic, Consultant Practitioner, Deputy Head of Clinical Care, Head of Operations.

Emergency Departments: ED Lead Matron, Divisional General/Operational Manager, ED Clinical Lead, Deputy Medical Director (Urgent and Emergency Care), Medical Director, ED Directorate Manager, EM Consultant, EM Clinical Director, Programme Manager (Unscheduled Care), Urgent and Emergency Performance Manager, Director of Operations (Medicine and Long Term Conditions), Deputy Divisional Director of Operations.

**Table D1.** Mapping of emergency departments that responded to the questionnaire to their partner ambulance services based on geography; level of ambulance handover delays for each ED and number of beds and bed occupancy in Winter 22/23.

| **Emergency department** | **Levels of ambulance handover delays** | **Partner AS** | **Number of beds** | **Bed occupancy (%)** |
| --- | --- | --- | --- | --- |
| ED1 | low | AS8 | >1500 | 85-90 |
| ED2 | low | AS3 | 0-500 | 90-95 |
| ED3 | low | AS9 | 500-1000 | 90-95 |
| ED4 | low | AS11 | 0-500 | >95 |
| ED5 | low | AS10 | 0-500 | 85-90 |
| ED6 | low | AS6 | 1000-1500 | 85-90 |
| ED7 | low | AS7 | 500-1000 | >95 |
| ED8 | high | AS5 | >1500 | 90-95 |
| ED9 | high | AS8 | >1500 | 85-90 |
| ED10 | high | AS6 | 500-1000 | 90-95 |
| ED11 | high | AS3 | 0-500 | >95 |
| ED12 | high | AS12 | 500-1000 | 85-90 |
| ED13 | high | AS11 | 500-1000 | >95 |
| ED14 | high | AS1 | 0-500 | 90-95 |
| ED15 | high | AS2 | >1500 | 90-95 |
| ED16 | high | AS10 | 1000-1500 | 90-95 |

**Table E1.** Total number of handovers in Winter period 22/23 for each ambulance service that responded to the questionnaire

| Ambulance service | **Total number of handovers in Winter 22/23** |
| --- | --- |
| AS1 | 28k-35k |
| AS2 | 28k-35k |
| AS3 | >35k |
| AS4 | >35k |
| AS5 | <28k |
| AS6 | <28k |
| AS7 | 28k-35k |
| AS8 | <28k |
| AS9 | >35k |
| AS10 | 28k-35k |
| AS11 | 28k-35k |
| AS12 | <28k |

| **F. Mapping of interventions to the SEIPS domains**  **Table F1.** Mapping of interventions to the SEIPS domains | | |
| --- | --- | --- |
| **SEIPS Domain** | **Ambulance Services** | **Emergency Departments** |
| **Tools and Technology** | **9/34 initiatives (26.5%)**   - ‘We created a visual management board showing free spaces for patients’ - ‘Our initiatives were co-designed by hospital and AS: traffic light systems, ambulance handover safety checklists and processes, escalation action cards, and communication channels.’ | **6/36 initiatives (16.7%)**   - ‘Single point of contact telephone line held by ED consultant to discuss potential conveyance of patients by ambulance with paramedic team with patient. Ambulance to contact the single point of access service here at the Trust to discuss Medical or Surgical patient before arriving.’ - ‘Shortened ambulance handover form on the ED electronic patient record.’ |
| **Tasks** | **25/34 initiatives (73.5%)**   - ‘Calls can be made via Consultant Connect allowing for Professional-to-Professional Call with ED consultant to assist with decision support.’ - ‘Timely Handover Process ‘THP’ – handing over after a certain amount of time has passed - 90 minutes.’ | **25/36 initiatives (69.4%)**   - ‘We have changed how we run our pitstop area, splitting the nurse in charge role and handover nurse role to enable us to be more efficient and split the workload.’ - ‘Ambulance staff put wrist bands on patients to assisting in identification for patient safety, patients re-directed to UTC are able to be identified.’ |
| **Person** | **21/34 initiatives (61.8%)**   - ‘Responsibilities of management (ROM) role – designated individuals to oversee delays. Tactical officer in each of 5 divisions.’ - ‘Additional skills for paramedics’ | **20/36 initiatives (55.6%)**   - ‘A nurse-led Rapid Assessment and Treatment Service (RATS) was created to front-load investigations (ECG, bloods, imaging) and treatments (cannulation, oral & iv medications, catheterisation) for ambulance arrivals.’ - ‘Emergency Physician in Charge (EPIC)/Emergency Nurse in Charge (ENIC) sitting patients in the waiting room who are suitable (and fall outside the ambulance service criteria for fit 2 sit) without dedicated handover to triage nursing staff. Oversight from EPIC for early interventions.’ |
| **Organisation** | **33/34 initiatives (97.1%)**   - ‘Change of ambulance triage nurse's role and position (closer to Hospital Arrival screen (HAS) screen)’ | **31/36 initiatives (86.1%)**   - ‘Introduction of an ED-staffed cohort to look after up to 6 patients in a corridor outside of the department. ‘IMPACT’ nurse is made available to take handover for up to 4 patients and those patients are nursed in the corridor (ED is not staffed to provide this service, it relies on staff being re-deployed from elsewhere in the hospital).’ - ‘Fit 2 Sit Policy. Swift identification of outflow cubicle/fit-2-sit spaces from offload bay. Two areas have been fitted with recliner chairs: 4 in the Minors area, and 4 in a room off Majors.’ |
| **Internal environment** | **16/34 initiatives (47.1%)**   - ‘Additional beds in wards’ - ‘Urgent Treatment Centre (UTC) located close to the ED handover bay’ | **21/36 initiatives (58.3%)**   - ‘Full capacity protocol enacted, whereby patients are moved to wards where a definite discharge has been identified and the discharge lounge is unable to accommodate.’ - ‘Transfer hub where patients awaiting departure from ED could wait transport.’ |
| **External environment** | **0/34 initiatives (0%)** | **3/36 initiatives (8.3%)**   - ‘Ambulance offload policy - states the principles used by ED staff in deciding which patient gets the next available Majors space.’ - Drop and go. Automatic handover at 45 minutes |
